# Differentiation of nerve fibers storing CGRP and CGRP receptors in the peripheral trigeminovascular system

**DOI:** 10.1186/1129-2377-14-S1-P89

**Published:** 2013-02-21

**Authors:** S Eftekhari, K Warfvinge, F Blixt, L Edvinsson

**Affiliations:** 1 Department of Clinical Sciences, Division of Experimental Vascular Research, Lund University, Lund, Sweden

## Background

The dura mater with the meningeal artery has since long been hypothesized to play an important role in migraine. It has been suggested that neuropeptides such as calcitonin gene-related peptide (CGRP) and substance P can activate dura mast cells leading to secretion of vasoactive, pro-inflammatory and neurosensitzing mediators, thereby contributing to migraine pathogenesis. Method:Immunofluorescence was used to study the detailed distribution of and its receptor components- calcitonin receptor-like receptor (CLR) and receptor activity modifying protein 1 (RAMP1)- in whole-mount rat dura mater, using a set of newly characterized antibodies. Their relation to each other, to mast cells, myelin, substance P, neuronal nitric oxide synthase (nNOS), pituitary adenylate cyclase-activating polypeptide (PACAP) and vasoactive intestinal peptide (VIP) were studied. In addition, we examined expression of CGRP and its receptor components in fresly isolated human dura vessels.

## Results

CGRP expression was found in thin fibers, while CLR and RAMP1 were expressed in thicker fibers. Double-staining of CGRP and the receptor components showed no co-localization. CLR and RAMP1 expression were found in cells, co-localized with mast cell tryptase. Double-staining with CGRP and MBP showed no co-localization. CLR and RAMP1 immunoreactive fibers co-localized with MBP and NF160/200. Substance P fibers co-expressed CGRP. nNOS and VIP expression was very limited and these fibers were distinct from the CGRP positive fibers. Few PACAP immunoreactive fibers co-localized with CGRP. No expression of functional CGRP receptor was observed in human mast cells.

## Conclusions

CGRP is expressed in un-myelinated fibers C-fibers. CLR and RAMP1 are instead expressed in myelinated fibers A-fibers. This supports the view that activation of C-fibers may locally cause release of CGRP, which could act on A-fibers, mast cells and vascular smooth muscle cells. Interestingly, CLR and RAMP1 expression was found in rat dura mast cells, however, human mast cells lack expression of functional CGRP receptor.

